# Long-Term Impact of Childhood Adversity on the Gut Microbiome of Nursing Students

**DOI:** 10.3390/ijerph21010068

**Published:** 2024-01-08

**Authors:** Negin Kazemian, Tony Zhou, Naveen Chalasani, Apurva Narayan, Jose Guillermo Cedeño Laurent, Hector A. Olvera Alvarez, Sepideh Pakpour

**Affiliations:** 1 School of Engineering, University of British Columbia, Kelowna, BC V1V 1V7, Canada; negin.kazemian@ubc.ca; 2 Department of Computer Science, University of British Columbia, Kelowna, BC V1V 1V7, Canada; tozhou@mail.ubc.ca (T.Z.); naveen15@student.ubc.ca (N.C.); apurva.narayan@ubc.ca (A.N.); 3 Department of Computer Science, University of Western Ontario, 1151 Richmond St., London, ON N6A 3K7, Canada; 4 Department of Electrical and Computer Engineering, University of Western Ontario, 1151 Richmond St., London, ON N6A 3K7, Canada; 5 Department of Environmental Health, Harvard T.H. Chan School of Public Health, Boston, MA 02115, USA; memo.cedeno@rutgers.edu; 6 Department of Environmental and Occupational Health and Justice, Rutgers School of Public Health, Piscataway, NJ 08854, USA; 7 School of Nursing, Oregon Health & Science University, Portland, OR 97239, USA; olveraal@ohsu.edu

**Keywords:** childhood adversity, depression in nursing students, stress in nursing students, sleep quality, gut microbiome

## Abstract

Adverse childhood experiences (ACEs) encompass negative, stressful, and potentially traumatic events during childhood, impacting physical and mental health outcomes in adulthood. Limited studies suggest ACEs can have short-term effects on children’s gut microbiomes and adult cognitive performance under stress. Nevertheless, the long-term effects of ACEs experienced during adulthood remain unexplored. Thus, this study aimed to assess the long-term effects of ACEs on the gut microbiota of adult nursing students. We employed a multidimensional approach, combining 16S rRNA sequencing, bioinformatics tools, and machine learning to predict functional capabilities. High-ACE individuals had an increased abundance of *Butyricimonas* spp. and *Prevotella* spp. and decreased levels of Clostridiales, and *Lachnospira* spp. *Prevotella* abundance correlated negatively with L-glutamate and L-glutamine biosynthesis, potentially impacting intestinal tissue integrity. While nursing students with high ACE reported increased depression, evidence for a direct gut microbiota–depression relationship was inconclusive. High-ACE individuals also experienced a higher prevalence of diarrhea. These findings highlight the long-lasting impact of ACEs on the gut microbiota and its functions in adulthood, particularly among nursing students. Further research is warranted to develop targeted interventions and strategies for healthcare professionals, optimizing overall health outcomes.

## 1. Introduction

**Figure 1:**
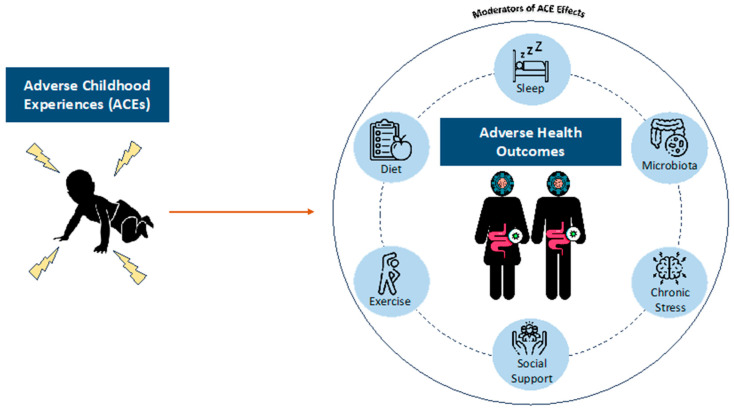
Factors moderating the effects of adverse childhood experiences (ACEs) on the mental and physical health of individuals.

Adverse childhood experiences (ACEs) encompass abuse (emotional, physical, and sexual), neglect, and household dysfunction [1,2,3,4]. Most adults have encountered at least one ACE [5], with prevalence at 65–87% [6,7]. ACE exposure has been associated with various health issues in adulthood, including cardiovascular disease [1,8], diabetes [1], cancer [9,10], depression [11,12], obesity, irritable bowel disease (IBD) [13], sexually transmitted infections [11,14], and premature mortality in adulthood [15]. An ACE history is also associated with risky behaviors such as smoking, inactivity, unsafe sexual practices [14,15], and substance use disorders [16]. Social, behavioral, and environmental factors may also moderate the effects of ACEs on the mental and physical health of individuals (Figure 1). Social support, for example, can buffer ACEs’ effects on health [17], but low socioeconomic status exacerbates stress, impacting sleep, activity, diet, and nutrition [18,19].

The gut microbiome, influenced by endogenous and exogenous factors and life events such as diet, age, stress, genetics, disease, and antibiotics, varies significantly among individuals [20,21]. The early years of childhood play a crucial role in shaping the gut microbiome, impacting biological and behavioral outcomes throughout life [22]. ACEs may also permanently affect the gut microbiome’s diversity and composition in adulthood [7,8,9]. For example, past studies of rodents have shown that early life stress can alter the gut microbiome [23,24]. Flannery et al. (2020) also demonstrated that caregiving behaviors can modify children’s gut microbiome, influencing both taxonomic and functional compositions [25]. In addition, a history of ACE may lead to impaired immune function [26,27,28] and dysregulated hypothalamic pituitary adrenal (HPA) axis function in adulthood [29,30]. Excitingly, emerging research has revealed the interconnectedness of the gut microbiota, the brain, and various aspects of the gut–brain axis [31,32]. In summary, the gut microbiome’s role in regulating immune homeostasis and its symbiotic relationship with the immune system suggests its potential role as a mediator in the ACE–neuroendocrine–immune function link [33,34,35].

The lifetime impacts of ACE can be even more critical for individuals with increased occupational stress [36]. Nursing is an occupation with an inherently increased exposure to stressors that start at nursing school. Nursing students, for example, often face a heavy academic workload, information overload, rigorous exams, and learning of complex skills in a fast-paced environment, along with long and irregular work hours while caring for patients in clinical settings [37,38]. Rella et al. (2009) conducted a study investigating fatigue and burnout among nursing students and found that 20% of graduates reported serious fatigue/stress and depression [39]. The presence of ACEs in their lives can exacerbate this risk even further, as studies have shown the association of high ACE with increased burnout severity and depressive symptoms among nursing students [40]. Additionally, research suggests that the gut microbiota may play a significant role in influencing the development and severity of depression [41,42,43,44]. For this reason, we hypothesized that the gut microbiota could exacerbate the impact of ACEs on nursing students’ mental health. Although research on stress among nursing students and nurses is abundant, no studies have examined the long-lasting impact of ACEs and considered the relationship between ACEs, the gut microbiota and its functions, and the overall health of nursing students. This insight could support gut–microbiome pathways to prevent depression-related challenges among nursing students, as these conditions may persist or worsen post-graduation and can contribute to increased healthcare costs and lower quality of healthcare [45,46,47]. 

To address these gaps, this study aimed to examine the impact of ACE exposure on the mental and physical health of nursing students. Specifically, we sought to determine whether ACE exposure has long-term effects on the gut microbiota and its function during adulthood. Lastly, we aimed to identify potential factors moderating the effects of ACEs on the gut microbiome, which could in turn serve as interventional targets to mitigate the lifelong consequences of ACE. 

## 2. Materials and Methods

### 2.1. Study Design and Participant Recruitment

This study was part of a larger prospective cohort study. Male and female participants (*n* = 150) were selected from an existing cohort of nursing students enrolled in the Bachelor of Science in Nursing (BSN) program at the University of Texas at El Paso (UTEP) who participated in the Nurse Engagement and Wellness Study (NEWS), a prospective cohort study examining how social, behavioral, and environmental factors may moderate the effects of stress experienced by nursing students and new nurses [48]. Media outlets, posters, flyers, emails, and information sessions held within classes were used to recruit eligible participants, and all participants provided written informed consent at the Biobehavioral Research Laboratory at UTEP. In addition, any participant who transferred to a different university, dropped out of the BSN program, did not graduate from the BSN program, was pregnant or lactating, or had been treated with antibiotics within the month prior to providing a stool sample was excluded from this study. This study was approved by review boards from the Harvard T.H. Chan School of Public Health (16-0080), UTEP (857149-1), and the University of British Columbia (UBC) (H21-03481). All research methods were performed in accordance with the relevant guidelines and regulations.

### 2.2. Stool Collection and Analyses

Sample collection took place from February to December 2018. Participants donated stool samples using a self-collected stool kit. This stool kit was modelled after the stool kit used by the Human Microbiome Project II and included two pre-labelled Para-Pak sampling vials. One Para-Pak vial was pre-filled with 5 mL of RNA*later* preservative. The second Para-Pak vial was pre-filled with 10 mL of 20% glycerol and 2 mL of acid-washed glass beads for bacterial strain isolation and analyses. Participants placed a teaspoon-sized sample of stool (approximately 1 g) into each sampling tube, stored the samples in a designated package within their personal freezer, and then delivered the package to the Biobehavioral Research Laboratory at UTEP within 24 h. Upon arrival at the lab, the stool samples were stored at −80 °C. Once all stool samples had been processed, the samples were stored on dry ice and shipped to the Massachusetts Host-Microbiome Center at Brigham and Women’s Hospital (MGH CCIB DNA core) for bacterial DNA extraction and sequencing. Following the extraction and purification of DNA from each stool sample, the V4 region of the 16S rRNA bacterial gene was amplified by polymerase chain reaction (PCR) using the universal bacterial primers 515F (forward primer) and 806R (reverse primer) as described previously [49,50]. Equal amounts of DNA from each purified PCR product were pooled together to generate an aggregated library for downstream processing. Illumina MiSeq sequencing was performed following standard Illumina MiSeq operation procedures. Demultiplexed, paired-end reads were generated for each sample using the default parameters of the Illumina MiSeq control software and imported into Quantitative Insights into Microbial Ecology (QIIME) version 2 (2021.8) [51]. A total of 10,202,879 raw reads were obtained, with an average of 68,019 reads per sample, and each forward and reverse read was 250 nucleotides in length. A minimum quality control score of 20 was selected: 13 bases were trimmed from the 5′ end of each read, and each read was truncated from the 3′ end at position *n* = 210 using the DADA2 denoise-paired method in QIIME 2 [52]. The resulting total frequency of amplicon sequence variants (ASVs) was 7,965,440, with an average frequency of 52,103 ASVs per sample. Taxonomy was assigned to each ASV using a pre-trained naïve Bayes classifier and the q2-feature-classifier plugin. This classifier was trained on the Greengenes 13_8 99% OTUs from the 515F/806R region of sequences [53]. Similar sequences were collapsed into single-replicate sequences, or operational taxonomic units (OTUs), to the genus level (Supplementary Table S1). Subsequently, taxa abundances were normalized by the total number of reads sequenced in each sample (Supplementary Table S1). All singletons and doubletons were removed from the normalized OTU table, which was then used to predict the microbiome’s biochemical pathways abundance (MetaCyc) using phylogenetic investigation of communities by reconstruction of unobserved states (PICRUSt2) (version 2.4.1) [54].

### 2.3. Blood Collection and Analysis 

Blood samples were also collected from participants. Serum and plasma were separated from the blood samples by centrifugation within an hour of collection and stored at −80 °C to preserve the integrity of the components for future analysis. Serum samples were then analyzed for levels of triglycerides, high-density lipoprotein cholesterol (HDL), low-density lipoprotein cholesterol (LDL), and glucose. Inflammatory markers were assessed via serum levels of C-reactive protein (CRP) and the inflammatory cytokines interleukin (IL)-1β, IL-6, IL-8, and tumor necrosis factor alpha (TNFα). Serum samples were analyzed in duplicate wells using the Milliplex™ MultiAnalyte Profiling (MAP) Human CVD Panel 3 pre-mixed kit (EMD Millipore Corp, Billerica, MA, USA) for CRP and the Human High-Sensitivity T Cell pre-mixed kit (EMD Millipore Corp, Billerica, MA, USA) for IL-1β, IL-6, IL-8, and TNFα. The plates were read on a Luminex 200 analyzer (Luminex Corporation, Austin, TX, USA) running Milliplex Analyst Version 5.1 software (Vigene Tech Inc. Carlisle, MA, USA) [48]. Concentrations for each biomarker were calculated with reference to a five-point best-fitting standard curve. 

### 2.4. Demographic and Collected Health Endpoints

Specific information pertaining to the different study assessments performed (health endpoints, biomarkers, life stress exposure, behaviors and personal traits, social factors, indicators of engagement and performance, and environmental exposure via clinical measures, biological sample collection, and self-reports) in the NEWS has been described in detail elsewhere [48]. In short, participants were required to complete a series of self-report questionnaires including a demographic questionnaire, the Adverse Childhood Experiences Questionnaire (ACE-Q), and the PHQ9 questionnaire. All questionnaires were completed anonymously in an online format during the first 4 weeks of the participants’ BSN program. Data from incomplete questionnaires were excluded from the analysis. Each participant also provided baseline demographic information including gender, race, ethnicity, and age (see Supplementary Table S1). 

### 2.5. Childhood Adversity

Upon arrival at the Biobehavioral Research Laboratory at UTEP, each participant completed the ACE-Q (approximately 20 min to complete on average) to assess exposure to adverse events that occurred before the age of 18, such as childhood maltreatment and neglect, household dysfunction, and abusive parenting [1]. The Centers for Disease Control and Prevention (CDC) have expanded the original seven-item ACE-Q to address the current ten central ACE categories recognized by the CDC. These ten ACE categories include three types of abuse, two types of neglect, and five types of family dysfunction [55]. ACE scores are calculated by adding the number of different ACE categories reported by an individual. Thus, ACE scores may range from 0 to 10 points and reflect the occurrence of an adverse event, not the severity or frequency of the event. ACE scores greater than or equal to 3 were considered “High ACE” and ACE scores less than 3 were considered “Low ACE” in this study. These parameters were based on the sample distribution.

### 2.6. Depression Questionnaires

Depression was measured using the patient health (PHQ9) questionnaire. The PHQ9 questionnaire (approximately < 10 min to complete) consists of the nine criteria on which the diagnosis of DSM-IV depressive disorder is based. Results of the PHQ-9 were scored as follows: a score of 5–9 was considered minimal depression; 10–15 was considered mild major depression; 15–19 was considered moderate major depression; and greater than or equal to 20 was considered severe major depression. 

### 2.7. Statistical Analysis

All collected and measured data were utilized to test whether any features were able to differentiate between ACE groups. Specifically, a dimensionality reduction model using principal component analysis (PCA) was used to compute the features which best described the principal components. The top features from this analysis were selected and, after checking normality (using the Shapiro–Wilk test), the Mann–Whitney U test was used to compare the features between ACE groups. All *p*-values less than 0.05 after false discovery rate (FDR) correction were considered significant.

Using 16S rRNA sequences, the Shannon diversity index for each sample was determined using the normalized OTU table with the singletons and doubletons removed and the Vegan: Community Ecology Package [56] within R (version 2021.09.1). The R packages ggplot2 [57], preseqR [58], and reshape2 [59] were used to construct a boxplot, and the t-test was performed to determine if any significant differences in alpha diversity existed within the data. Differences among bacterial community structures between samples (beta diversity) were determined using Bray–Curtis Dissimilarity from the Vegan: Community Ecology Package within R (version 4.0.3) [56]. Significant differences in beta diversity were evaluated using the ANOSIM test, and any *p*-value less than 0.05 was considered significant. 

To test whether certain gut microbiota and functional biochemical pathways were predictive of ACE, a random forest (RF) model [60] was trained on high- and low-ACE samples at the genus level, and again separately for the biochemical pathways using the Python library Scikit-learn [61]. A dimensionality reduction model was utilized to ensure that the trained RF model avoided overfitting and generalized better on the data [62]. PCA was used to compute the features which best described the principal components. The top twenty features from this analysis were selected and used in the training process for the RF model. The leave-one-out (LOO) cross-validation method was then implemented to assess how effectively the trained classifier generalized in the event of unseen data. All measured test errors were averaged to determine the cross-validation error value. The receiver operating characteristic (ROC) curve was plotted for each LOO dataset. Then, Fisher’s exact statistical significance test was utilized to compare the RF classifiers with the greatest validation scores and determine the differences between the predictive performances of the top RF candidates [63]. The RF model was run 100 times, and the average mean decrease in impurity (MDI) of the most notable features was determined [64]. The linear discriminant analysis (LDA) effect size (LEfSe) method was also performed to identify key taxa and biochemical pathways that may contribute to the observed differences in gut microbial communities using relative abundances [65]. The LEfSe algorithm coupled the non-parametric Kruskal–Wallis test and the unpaired Wilcoxon test with LDA scores to estimate the LDA effect sizes of differentially abundant bacterial OTUs and functional biochemical pathways with statistical significance [65]. The overlapping microbial taxa and biochemical pathways from RF and LEfSe analysis were then used for further analysis and comparisons between groups. Using multiple machine-learning algorithms and finding consensus between their results increases the reliability, generalization, and robustness of the analysis, reducing bias and overfitting. It allows for model selection, performance comparison, and more accurate insights into the underlying patterns and relationships within the data. Gut microbiomes and biochemical pathways were considered to be significantly different if the *p*-value was less than 0.05 and if the LDA score (log10) threshold was greater than 3.0.

Lastly, network analysis was utilized to assess the relationships between various features differentiating low- and high-ACE groups with potential factors that could moderate the effects of ACEs. The k-nearest neighbor (KNN) algorithm based on Euclidean distance was used to estimate and substitute missing data (*k* = 5) [66]. The network was constructed based on Spearman’s rank correlation coefficients whose rho values were more than 0.3 and *p*-adjusted < 0.05 (using FDR). 

## 3. Results

### 3.1. Demographic Characteristics and Features Differentiating ACE Groups

This study included a total of 150 adult male and female nursing students who were enrolled in the BSN program at UTEP and members of the original NEWS cohort [48]. The average participant’s age was 26 years (SD = 6.5), and the average BMI score was 26.3 (SD = 5.6) (Table 1). The majority of the study participants self-identified as female (83%), Hispanic (87%), and White (88%) (Table 1). Furthermore, 53% of the participants reported “High ACE” scores (≥3), and 47% of the participants reported “Low ACE” scores (<3) (Table 1). 

**Figure 2:**
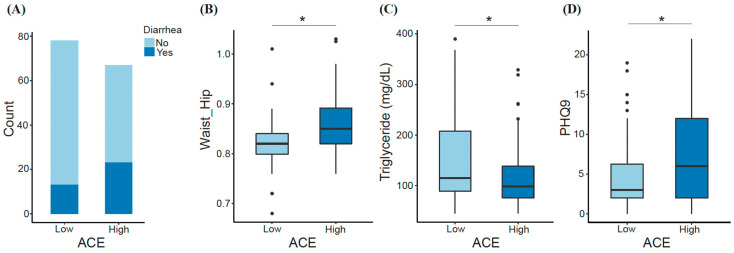
Significant features differentiating the low- and high-ACE groups. Factors including (**A**) diarrhea episodes within two weeks prior to sample collection, (**B**) waist-to-hip ratio, (**C**) triglyceride (mg/dL), and (**D**) depression index (PHQ9) were compared between ACE groups. Significant differences were determined using the Mann–Whitney U test and FDR correction. * *p*-adjusted < 0.05. ACE, adverse childhood experience.

We observed some key differences between low- and high-ACE groups. For instance, having experienced diarrhea within the last two weeks prior to sample collection was among one of the factors differentiating ACE groups. Diarrhea was experienced by 16.7% of the low-ACE group compared with 34.4% of the high-ACE group (Figure 2A). Also, triglycerides were significantly decreased in individuals with high ACE (Mann–Whitney U; *p*-adjusted = 0.03), while waist-to-hip ratio (Mann–Whitney U; *p*-adjusted = 0.024) and depression (Mann–Whitney U; *p*-adjusted = 0.025) were significantly increased in individuals with high ACE (Figure 2B–D). Inflammatory markers were not significantly different between ACE groups.

### 3.2. Gut Microbiome Diversity Analysis

**Figure 3:**
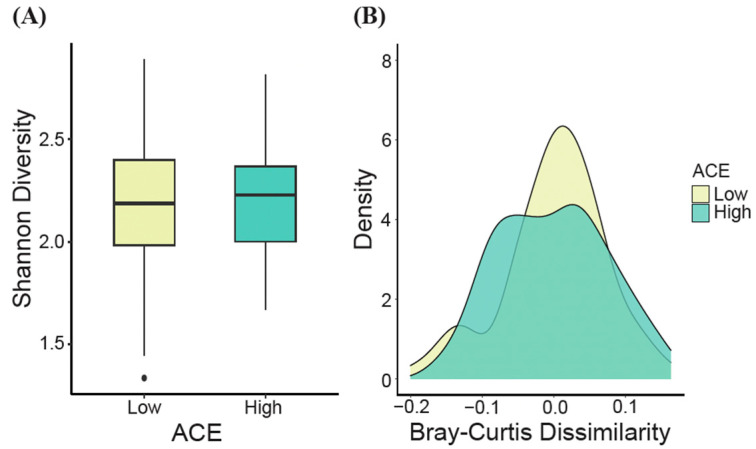
Gut microbial diversity (genus level). (**A**) The alpha diversity (Shannon’s diversity index) and (**B**) the beta diversity density plot (Bray–Curtis dissimilarity) for ACE groups were determined. Significant differences were determined using the t-test and ANOSIM for alpha and beta diversity, respectively. ACE, adverse childhood experience.

At the genus level, no significant differences in alpha diversity, as measured by the Shannon diversity index, were observed between the ACE groups (*t*-test, *p* > 0.05, Figure 3A). However, when generating density plots based on the estimated Bray–Curtis dissimilarity values, significant differences in beta diversity were detected for the ACE groups (ANOSIM, *p* = 0.04, Figure 3B). 

### 3.3. Bacteria Differentiating ACE Groups

**Figure 4:**
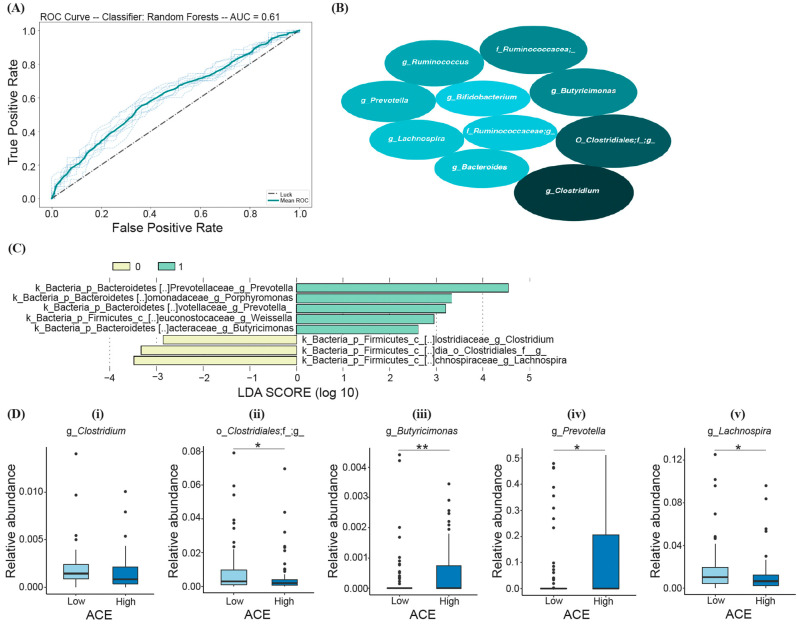
RF and LEfSe analysis characterizing the significant bacterial taxa in the high- and low-ACE groups. The implemented RF model led to (**A**) the ROC curve displaying an AUC of 61% (*p* > 0.05). (**B**) The top ten most important bacteria differentiating ACE groups, where the size of the ellipses represents the average mean decrease in impurity (MDI). (**C**) Histogram of the LDA scores (log10) calculated for taxa with differential abundances in high- and low-ACE individuals. The high-ACE group is represented in green (1.0) and the low-ACE group is represented in yellow (0.0). (**D**) Overlapping bacterial taxa from both random forest and LEfSe compared between ACE groups using the Mann–Whitney U test and FDR correction. * *p*-adjusted < 0.05; ** *p*-adjusted < 0.01. ACE, adverse childhood experience.

Based on the RF model, the following top ten most important bacteria were identified to differentiate ACE groups (listed in the order of decreasing average mean decrease in impurity (MDI)): *Clostridium* spp., members of Clostridiales, *Butyricimonas* spp., *Ruminococcacea.*, *Ruminococcus* spp. *Prevotella* spp., *Lachnospira* spp., *Bacteroides* spp., members of Ruminococcaceae, and *Bifidobacterium* spp. (Figure 4B); however, the prediction model was determined to be not significant (Fisher’s exact test; *p* > 0.05) (Figure 4A).

LEfSe analysis was also performed in comparison with RF to test which groups of bacteria differentiate low- and high-ACE individuals and are predictive of ACE. From the calculated LDA scores, certain taxa were identified for the “High ACE” group (*Prevotella*, *Porphyromonas, Weissella*, and *Butyricimonas*), and three taxa were identified for the “Low ACE” group (*Lachnospira*, *Clostridiales*, and *Clostridium*) (Figure 4C). The taxa with the greatest relative abundance (characterized by the LDA score with the highest magnitude) were the genera *Prevotella* and *Lachnospira* for the high- and low-ACE groups, respectively (Figure 4C). 

Random forest and LEfSe analysis revealed five overlapping taxa of bacteria that can differentiate between ACE groups, including *Clostridium*, Clostridiales, *Butyricimonas*, *Prevotella*, and *Lachnospira* (Figure 4D). High-ACE individuals exhibited decreased levels of Clostridiales (Mann–Whitney U, *p*-adjusted = 0.042, Figure 4D-ii) and *Lachnospira* (Mann–Whitney U, p-adjusted = 0.041, Figure 4D-v), as well as increased levels of *Prevotella* (Mann–Whitney U, *p*-adjusted = 0.043, Figure 4D-iv) and *Butyricimonas* (Mann–Whitney U, *p*-adjusted = 0.008, Figure 4D-iii). However, there was no significant difference in *Clostridium* levels between the two groups after correction (Mann–Whitney U, *p*-adjusted > 0.05, Figure 4D-i).

### 3.4. Functional Differences between ACE Groups

**Figure 5:**
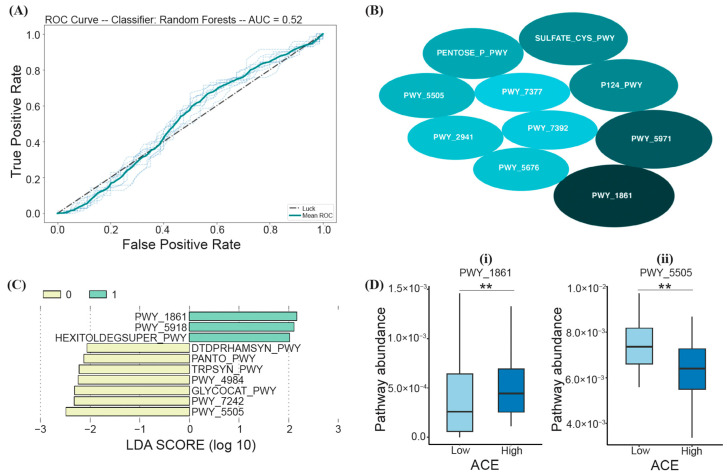
RF and LEfSe analysis characterizing the significant biochemical pathways (via PICRUSt2) in the high- and low-ACE groups. The implemented RF model led to (**A**) the ROC curve displaying an AUC of 52% (*p* > 0.05), (**B**) the top ten most important pathways differentiating ACE groups, where the size of the ellipses represents the average mean decrease in impurity (MDI), (**C**) a histogram of the LDA scores (log10) calculated for pathways with differential abundances in high- and low-ACE individuals (the high-ACE group is represented in green (1.0), and the low-ACE group is represented in yellow (0.0)), and (**D**) overlapping pathways from both RF and LEfSe compared between ACE groups using the Mann–Whitney U test and FDR correction. ** *p*-adjusted < 0.01. ACE, adverse childhood experience.

Based on the RF model trained on samples from both high- and low-ACE individuals, a PCA identified the top ten most important biochemical pathways that may predict ACE (listed in the order of decreasing average MDI), including PWY_1861 (formaldehyde assimilation II-assimilatory RuMP Cycle), PWY_5971 (palmitate biosynthesis II), P124_PWY (*Bifidobacterium* shunt), SULFATE-CYS-PWY (sulfate assimilation and cysteine biosynthesis), PENTOSE_P_PWY (the pentose phosphate pathway), PWY_5505 (L-glutamate and L-glutamine biosynthesis), PWY_2941 (L-lysine biosynthesis II), PWY_5676 (acetyl-CoA fermentation to butanoate), PWY_7392 (taxadiene biosynthesis), and PWY_7377 (cob(II)yrinate a,c-diamide biosynthesis I) (Figure 5B); however, based on LOO cross-validation, the prediction model was not significant (Fisher’s exact test; *p* > 0.05) (Figure 5A). 

LEfSe analysis was also performed to assess the functional differences between low- and high-ACE individuals. From the calculated LDA scores, three functional pathways were identified for the “High ACE” group (PWY_1861 (the formaldehyde assimilation II-assimilatory RuMP cycle), PWY_5918 (the superpathway of heme b biosynthesis from glutamate), and HEXITOLDEGSUPER_PWY (the superpathway of hexitol degradation (bacteria))) (Figure 5C). Seven functional pathways were identified for the “Low ACE” group (DTDPRHAMSYN_PWY (dTDP-L-rhamnose biosynthesis), PANTO_PWY (phosphopantothenate biosynthesis I), TRPSYN_PWY (L-tryptophan biosynthesis), PWY_4984 (the urea cycle), GLYCOCAT_PWY (glycogen degradation I), PWY_7242 (D-fructuronate degradation), and PWY_5505 (L-glutamate and L-glutamine biosynthesis)) (Figure 5C). The functional pathways with the greatest relative abundance (characterized by the LDA scores with the highest magnitude) were PWY_1861 and PWY_5505 for the high- and low-ACE groups, respectively (Figure 5C). 

Random forest and LEfSe analysis led to two overlapping pathways that can differentiate between ACE groups, PWY_1861 and PWY_5505 (Figure 5D). High-ACE individuals had decreased PWY_5505 (L-glutamate and L-glutamine biosynthesis; Mann–Whitney U; *p*-adjusted = 0.01) and increased PWY_1861 (formaldehyde assimilation II-assimilatory RuMP Cycle; Mann–Whitney U; *p*-adjusted = 0.01) (Figure 5D). 

Lastly, using network analysis (rho > 0.3 or rho < −0.3), we assessed the relationships between various features differentiating the low- and high-ACE groups (i.e., bacterial taxa, biochemical pathways, diarrhea, depression, waist-to-hip ratio, and triglycerides), with potential factors that could moderate the effects of ACEs (i.e., sleep, diet, and physical activity). We found that the bacterial genus *Prevotella* was negatively correlated with L-glutamate and L-glutamine biosynthesis (PWY-5505) (Spearman correlation; rho = 0.33; *p*-adjusted = 0.001), with no significant correlations with potential moderators. On the other hand, decreased sleep quality was found to be significantly related to depression (Spearman correlation; rho = 0.43; *p*-adjusted = 0.001), but not linked to any affected bacterial taxa or functions. 

## 4. Discussion

In our quest to investigate the long-lasting impacts of ACEs, we examined the gut microbiota and its functions in adulthood. We observed distinct differences between individuals with low and high ACE exposure in gut microbiota composition and the relative abundance of specific bacterial taxa. Notably, high-ACE individuals displayed an increased relative abundance of *Butyricimonas* spp. and *Prevotella* spp. while exhibiting decreased levels of Clostridiales and *Lachnospira* spp. These observations align with a previous study where pregnant women with a history of high ACE showed a higher abundance of *Prevotella* [35]. It is worth noting that an overabundance of certain *Prevotella* spp. has been associated with autoimmune diseases, chronic inflammatory conditions, hypertension, and heart failure [67,68,69,70], suggesting potential health implications of ACE-related microbial changes. Furthermore, we observed a negative correlation between *Prevotella* abundance and L-glutamate and L-glutamine biosynthesis, which were found to be decreased in the high-ACE group. Glutamine plays a vital role in maintaining intestinal tissue integrity by promoting enterocyte proliferation and inducing the expression of tight-junction proteins [71]. Depletion of glutamine stores may occur during trauma, sepsis, and inflammatory bowel disease (IBD) [71], providing a potential link between stressful events in childhood and decreased gut health later in life. 

The higher prevalence of diarrhea among individuals in the high-ACE group suggests that changes in gut microbiota, particularly the increased abundance of *Prevotella*, may play a key role in gut health across the lifespan. *Prevotella* contributes to inflammation by producing lipopolysaccharide (LPS), which can trigger Toll-like receptor 4 (TLR-4), leading to low-grade inflammation and diarrhea-predominant irritable bowel syndrome (IBS) [72,73]. Increased diarrhea can also be attributed to the effects of stress on the gastrointestinal system. For instance, when the stress hormone corticotropin-releasing hormone (CRH) was injected into the hypothalamus of rats, it resulted in a significant 84% reduction in colonic transit time [74]. Consequently, this accelerated passage of stool through the colon can lead to greater excretion of water and nutrients from the body, potentially contributing to an increase in diarrhea episodes [75]. Hence, the role of diarrhea in the connections between ACEs, the gut microbiota, and its functions warrants further investigation.

We also observed increased depression among nursing students with high ACE, further highlighting the potential to explore the gut–brain axis in individuals with ACEs. Previous research has also shown a relationship between ACE and depression among nursing students [40]. A study performed by Chapman et al. (2004) observed a strong positive relationship between ACE score and the likelihood of developing chronic depressive disorder in adulthood [12]. In addition, previous research has suggested a potential relationship between the gut microbiome and depression, indicating that exploring the gut–brain axis may shed light on the mechanisms underlying the association between gut health and depressive disorders. For example, in a study examining the impact of ACE and caregiver stress on the gut microbiota in children (ages 5–7), researchers discovered that the microbial composition of the gut was significantly altered by socioeconomic risk, dysfunction in the relationship between the caregiver and the child, and dysregulation in the child’s behavioral patterns [25]. The increased level of *Butyricmonas* and reduced levels of Clostridiales have also been associated with stress [76,77] and depression [78]. However, contrary to previous studies, our findings did not reveal a direct relationship between the gut microbiome and depression. This could be because ACEs may also affect the overall adaptiveness of the body’s immune response and lead to increased levels of pro-inflammatory molecules [27,79]. Gut microbiota metabolites, such as neuropeptides and short-chain fatty acids (SCFAs), play a key role in the gut–brain axis by interacting with the central nervous system, activating microglia, which modulate HPA activity and, in turn, cytokine release [80]. Interestingly, multiple rodent studies have revealed that exposure to early life stress (ELS), the functional equivalent of ACE in rodents, has been linked to increased intestinal permeability, bacterial translocation across the gut epithelial barrier, and alterations in the composition of the gut microbiota [81,82,83]. These changes may elicit an immune response and affect the overall body system. Therefore, further exploration of the connections between ACEs and the gut–brain axis may provide valuable insights into the mechanisms through which stress in childhood affects health and well-being across the lifespan.

Lastly, our study explored potential moderators that might mitigate the lasting health effects of ACEs. Improving sleep quality may help combat depression among nursing students with high ACE, given the well-established link between depression and sleep disturbances [84,85]. Sleep disturbances can have wide-ranging effects on gastrointestinal, psychiatric, and neurological health [86,87,88]. Notably, sleep quality has also been found to influence the gut microbiota, with circadian clock misalignment and sleep deprivation affecting the microbial community structure in the gut [89,90]. Maltreated children, who are at an elevated risk of sleep problems, may also experience increased inflammation levels and metabolic disruptions in the gut, significantly impacting overall health [91]. Therefore, enhancing sleep quality could serve as an intervention for high–ACE individuals, potentially reducing depressive symptoms and improving overall health. 

This research is the first study investigating the long-term effects of ACES on the gut microbiota and its associated functions in adult nursing students. Our comprehensive methodology allowed us to gain a holistic understanding of the impact of ACEs on the gut microbiota and its potential implications for nursing students’ well-being. The limitations of the study include the low racial and ethnic diversity of the student cohort, as 88% of the participants self-identified as White and 87% self-identified as Hispanic. Thus, additional studies with an increased number of participants and diverse demographic characteristics are needed to provide a more accurate representation of the general student population of the world. Furthermore, instances of ACEs are self-reported on the ACE questionnaire. Since everyone responds differently to chronic stress and trauma, instances of ACEs may be over- or under-reported on the ACE questionnaire. Therefore, ACE scores can only be used to demonstrate correlations or associations between childhood adversity and the gut microbiome and functional changes in adulthood. Future studies are encouraged to incorporate a longitudinal approach with the collection of comprehensive health histories, specifically of gastrointestinal disorders, to better understand the long-term impacts of ACEs. In addition, detailed data collection can serve as an extensive control for potential confounding factors in order to address the complex landscape of microbiome assessments. It is crucial to acknowledge that further research and comprehensive assessments are needed to fully understand the intricate interplay between ACE exposure, the gut microbiome, and various adverse health and mental health outcomes in adulthood.

## 5. Conclusions

In conclusion, this study substantiates that adult nursing students with high ACE display a notable alteration in gut microbiotal composition and functionality, with implications for intestinal health and potential associations with psychological well-being. Focusing on functional interactions within the gut microbiome and the metabolites produced provides a more comprehensive understanding of the intricate mechanisms underlying the relationship between ACE and adverse health outcomes. Such understanding can help inform potential therapeutic strategies to restore microbial balance and promote intestinal health in individuals with high ACE. Probiotic use may offer a promising approach to alleviate diarrhea symptoms associated with ACEs and improve overall gut health. Through further exploration of the gut–brain axis and potential interventions, we can pave the path for future healthcare approaches catered to individuals with high ACE, ultimately improving their well-being and quality of life. Collaboration and ongoing research are vital in fully unravelling ACE’s impact and devising effective strategies to promote optimal health for all those affected by early-life adversity.

## Figures and Tables

**Table 1:** Summary of the sample population.

	Dimension	*n*	Mean (%)	SD
Age	-	150	26	6.5
BMI	-	150	26.3	5.6
Gender	Male	26	(17)	-
	Female	124	(83)	-
Ethnicity	Hispanic	130	(87)	-
	Non-Hispanic	20	(13)	-
Race	American Indian	5	(3)	-
	Asian	6	(4)	-
	African American	7	(5)	-
	Native Hawaiian	0	(0)	-
	White	132	(88)	-
ACE	Low (<3)	80	(53)	-
	High (≥3)	70	(47)	-

*n,* number of students per group; SD, standard deviation; BMI, body mass index; ACE, adverse childhood experience.

## Data Availability

The metadata and normalized and non-normalized feature tables are available in the supplementary data. The raw 16S rRNA sequence data are available at https://www.ncbi.nlm.nih.gov/sra/PRJNA1024544 (accessed on 7 January 2024).

## Supplementary Materials

The following supporting information can be downloaded at: https://www.mdpi.com/article/10.3390/ijerph21010068/s1, Table S1: Study metadata and normalized and non-normalized feature tables.
